# Conceptualising patient empowerment: a mixed methods study

**DOI:** 10.1186/s12913-015-0907-z

**Published:** 2015-07-01

**Authors:** Paulina Bravo, Adrian Edwards, Paul James Barr, Isabelle Scholl, Glyn Elwyn, Marion McAllister, the Cochrane Healthcare Quality Research Group, Cardiff University

**Affiliations:** Institute of Cancer & Genetics, Cardiff University, Heath Park, Cardiff, CF14 4XN UK; School of Nursing, Pontificia Universidad Católica de Chile, Avenida Vicuña Mackenna 4860, Macul, Santiago Chile; Cochrane Institute of Primary Care & Public Health, Neuadd Merionnydd, Cardiff University, Heath Park, Cardiff, UK CF14 4YS UK; The Dartmouth Center for Health Care Delivery Science, Dartmouth College, 37 Dewey Field Road, 4th Floor, Hinman Box 7256, Hanover, NH 03755 USA; Department of Medical Psychology, University Medical Center Hamburg-Eppendorf, Hamburg, Germany

**Keywords:** Patient empowerment, Definition, Conceptual map, Scoping study, Framework analysis, Thematic analysis

## Abstract

**Background:**

In recent years, interventions and health policy programmes have been established to promote patient empowerment, with a particular focus on patients affected by long-term conditions. However, a clear definition of patient empowerment is lacking, making it difficult to assess effectiveness of interventions designed to promote it. The aim in this study was to develop a conceptual map of patient empowerment, including components of patient empowerment and relationships with other constructs such as health literacy, self-management and shared decision-making.

**Methods:**

A mixed methods study was conducted comprising (i) a scoping literature review to identify and map the components underpinning published definitions of patient empowerment (ii) qualitative interviews with key stakeholders (patients, patient representatives, health managers and health service researchers) to further develop the conceptual map. Data were analysed using qualitative methods. A combination of thematic and framework analysis was used to integrate and map themes underpinning published definitions of patient empowerment with the views of key UK stakeholders.

**Results:**

The scoping literature review identified 67 articles that included a definition of patient empowerment. A range of diverse definitions of patient empowerment was extracted. Thematic analysis identified key underpinning themes, and these themes were used to develop an initial coding framework for analysis of interview data. 19 semi-structured interviews were conducted with key stakeholders. Transcripts were analysed using the initial coding framework, and findings were used to further develop the conceptual map. The resulting conceptual map describes that patient empowerment can be conceived as a state ranging across a spectrum from low to high levels of patient empowerment, with the level of patient empowerment potentially measurable using a set of indicators. Five key components of the conceptual map were identified: underpinning ethos, moderators, interventions, indicators and outcomes. Relationships with other constructs such as health literacy, self-management and shared decision-making are illustrated in the conceptual map.

**Conclusion:**

A novel conceptual map of patient empowerment grounded in published definitions of patient empowerment and qualitative interviews with UK stakeholders is described, that may be useful to healthcare providers and researchers designing, implementing and evaluating interventions to promote patient empowerment.

**Electronic supplementary material:**

The online version of this article (doi:10.1186/s12913-015-0907-z) contains supplementary material, which is available to authorized users.

## Background

Empowerment has gained prominence in healthcare, as part of a move away from paternalism towards more equitable and collaborative models of healthcare delivery [1–5], with the potential for improving cost-effectiveness of care, especially for people affected by long term conditions (LTCs) [6–8]. However, the capacity of existing outcome measures to capture the patient benefits of these interventions has been questioned [9]. Management of LTCs is a significant challenge facing healthcare systems worldwide [10]. Consequently, the National Health Service (NHS) in England initiated a cross-governmental strategy for tackling LTCs [11], including a mandate to involve people in making decisions about their own care [12]. This is likely to include developing primary care interventions to empower patients to self-manage LTCs, since this is a key aspect of policy strategies to control healthcare costs [13–15].

In Europe, patient empowerment is supported by the EU through the European Patients Forum which aims to “… promote the development and implementation of policies, strategies and healthcare services that empower patients to be involved in the decision-making and management of their condition…”. In the UK, “High quality care for all” [15] committed the NHS to patient empowerment by (1) giving patients more choice and control over their healthcare (2) making hospital funding contingent upon performance against a range of quality measures including patient reported outcomes measures (PROMs) (3) implementing use of personalized care plans and personal health budgets. NHS England’s Five Year Forward View (October 2014) reiterated a commitment to patient empowerment, to be enacted through shared decision-making and novel mechanisms such as “integrated personal commissioning”, a new approach involving blended health and social care funding for people with complex needs [16]. It has been proposed that healthcare services, interventions and policy implementations for LTCs can be evaluated using PROMs designed to capture patient empowerment [2]. PROMs are short self-completion questionnaires designed to capture aspects of patient health status or health-related quality of life reported directly by the patient without any interpretation by a clinician. However, patient empowerment is neither well-defined nor consistently operationalised and there is no consensus on the best way to measure it [1, 2]. Some validated condition-specific questionnaires that capture empowerment include the Empowerment Scale (mental health) [9], the Diabetes Empowerment Scale [17], and the Patient Empowerment Scale (cancer) [18]. This is not an exhaustive list, but each of these questionnaires was developed independently using a different definition of patient empowerment. There are also some generic validated questionnaires that capture similar constructs, but that do not claim to capture patient empowerment e.g., The Patient Activation Measure, which captures ability and willingness of patients to manage their own health and health care [19] and the Patient Enablement Instrument, which captures patients’ capacity to understand and cope with their health issues [20].

As a result, approaches, interventions and policies in healthcare are often not clear in what they intend to achieve and therefore cannot be evaluated or compared on the basis of how effectively they empower patients. Some conceptual clarity is needed to enable focused patient empowerment interventions to be developed and evaluated, and to facilitate selection of appropriate PROMs to use across a range of instances where patient empowerment is an implicit or explicit goal (shared decision-making, self-care/self-management programmes, integrated personal commissioning) to evaluate whether that goal has been achieved. The aim of this research was to develop a conceptual map of patient empowerment, identifying components of patient empowerment and relationship with other constructs e.g., health literacy, self-management and shared decision-making, with a focus on primary care because many patients affected by LTCs primarily use healthcare services in this context.

## Methods

**Figure 1:**
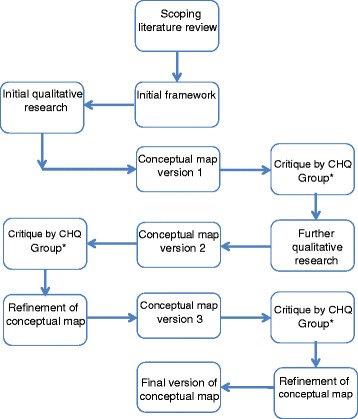
Iterative process of developing the conceptual map

A mixed methods study (see Fig. 1) involving a scoping literature review [21] and qualitative interviews was conducted to identify the components of patient empowerment and develop a conceptual map of patient empowerment. The developing conceptual map was subject to regular critique by members of the Cochrane Healthcare Quality Research Group, Cardiff University, a group of health researchers specialising in healthcare quality, patient centred care, shared decision making, and health literacy, contributing to further development of the conceptual map.

### Scoping literature review

A scoping literature review is a method that can be used to ‘map’ relevant key concepts in the literature underpinning a research area [20]. The scoping literature review followed the five-stage framework proposed by Arksey and O’Malley [21], which were to 1) identify the research question; 2) identify relevant studies; 3) select studies; 4) chart the data; and 5) collate, summarise and report the results. The research question was: “What key concepts underpin published definitions of patient empowerment?” The following sources were searched in February 2013 for articles published in English over the previous five-year period using the terms (Patient) AND (Empowerment): Ovid Medline, Scopus, CINAHL Plus, EMBASE and PsyINFO. Articles were included if they contained the searched terms in the title or abstract only. All types of articles published in peer-reviewed journals were considered eligible. All titles and abstracts available were first evaluated for relevance by two authors (PBr & MM). Following Makoul and Clayman’s method for conceptualisation [22], all citations which included a definition of patient empowerment were recorded. This enabled identification of the frequency of previously published articles referenced by each definition.

Data were extracted under the following headings: research identification (authors, year of publication, country of study sample, and study population), research methods and the definition or description of patient empowerment used. Articles that included a definition of patient empowerment were read by one member of the research team (PBr) who marked and extracted each section where a definition/description of patient empowerment was provided. Data extracted were stored in tables and initially analysed by two team members (PBr & MM) independently to identify conceptual themes underpinning the extracted definitions of patient empowerment to identify components of patient empowerment. Data were analysed using thematic analysis [22], which involves the following five steps: (i) familiarisation with the data (ii) generation of initial codes from the data using an inductive approach (ii) searching for candidate themes by sorting and collating codes into broader themes (iii) refining candidate themes and developing an initial map of patient empowerment components (iv) defining and naming patient empowerment components, once a satisfactory map of the data has been developed. In a second step both researchers agreed on identification of patient empowerment components, and a conceptual map of patient empowerment was drafted.

### Interviews with key stakeholders

The aim was to recruit a purposive sample consisting of four patients, four patient representatives, four primary care clinicians, four health managers and four health service researchers to share their views on patient empowerment in qualitative interviews. Ethical approval was granted by the School of Medicine at Cardiff University and the UK NHS National Research Ethics Service (Ref. 13/SC/0190). Patients and patient representatives were contacted through patient organisations in Wales. Clinicians, health managers and researchers were approached through professional networks in Wales and England. Inclusion criteria were that participants had to be adults aged 18 or over who have personal/family experience of LTCs, or who have professional or health service research experience with people affected by LTCs. A purposive sample was designed in an attempt to maximize diversity of responses.

Two semi-structured interviews were designed, one for patients and patient representatives and one for clinicians, health managers and health researchers (Tables 1 and 2 respectively). Following approved informed consent procedures, semi-structured interviews were conducted [23], using the same questions within a flexible framework [24]. Interviews took place in person or by telephone, and were audio-recorded and transcribed in full. Using the conceptual map developed from the literature review as an initial ‘framework’, interview transcripts were analysed using the five step approach developed by Ritchie & Spencer [25, 26] to further develop the conceptual map. This method is more structured than many other qualitative approaches, and involves development of a working analytical framework that is then used to index the data, whilst remaining sufficiently flexible in the early stages to allow incorporation of additional themes [27]. These five steps are: (i) familiarisation with the data (ii) (further) development of a thematic framework by identifying all key themes (iii) indexing all the data in textual form by coding the transcripts, supported by short text descriptors for each code (iv) charting or classifying the data according to the relevant part of the thematic framework to which they relate and finally (v) using charts or diagrams to map the themes identified, including relationships between themes. In this phase of the analysis, mapping and interpretation of data were influenced both by the original research question and by the patient empowerment components identified in the interview transcripts. Assisted by the computer software ATLAS.ti 6, one researcher conducted the analysis (PBr). To ensure reliability of coding [28], a second researcher (MM) independently coded 25 % of transcripts, which were discussed with PBr to agree a coding framework. See Additional file 1 for RATS Checklist.

**Table 1:** Semi-structured interview guide for patients and patient representatives

Topic	Question	Probes
Patient outcomes	1) What difficulties have you (or the people you represent) experienced in your/their everyday/family life due to this condition?	- What were you/they not able to do in your/their day-to-day life because of the condition?
	2) If you think about a time when you (or the people you represent) used the primary care service, what benefits did they get from this?	- How did the condition limit what you/they could do, and how did the care you/they received make a difference to that?
	3) What do you think people are looking for when they see the GP/use primary care services? What do they want to get out of it?	- In what way(s) was your/their life improved afterwards?
		- What helped you/them to get what you/they needed/wanted?
		- What prevented you/them them from getting what you/they needed/wanted?
	4) Has anything changed for you/your family/the patients you represent as a result of your/their use of primary care services?	- Can you say more about what those improvements are?
	5) Are there any ways in which your life/your family life/the family lives of the patients you represent became more difficult following your/their use of primary care services?	- Can you say more about what those difficulties are?
Patient empowerment as a measurable outcome	6) What does the term “patient empowerment” mean to you?	- Please describe health interventions or health services that promote patient empowerment.
	7) Would a patient questionnaire capturing the degree of patient empowerment be useful to assess the quality of primary care services?	- What sorts of practical things would make it hard to use a patient questionnaire capturing patient empowerment effectively to assess the quality of primary care services?
		- What sorts of practical things would support use of such a questionnaire?

**Table 2:** Semi-structured interview guide for clinicians, health managers and health researchers

Topic	Question	Probes
Patient outcomes	1) What do you think people are looking for when they see the GP/use primary care services? What do they want to get out of it?	- What helps patients to get what they need/want from primary care services?
		- What prevent patients from getting what they need/want from primary care services?
	2) What patient reported outcomes do you think are useful to assess quality in primary care?	- How is it measured at the moment?
		- Are these approaches effective?
		- Are there any other measures that could be be considered?
Patient empowerment as a measurable outcome	3) What does the term "patient empowerment" mean to you?	- Please describe health interventions or health services/approaches that promote patient empowerment.
	4) Would a patient questionnaire capturing the degree of patient empowerment be useful to assess the quality of primary care services?	- What sorts of practical things would make it hard to use a patient questionnaire capturing patient empowerment effectively to assess the quality of primary care services?
		- What sorts of practical things would support use of such a questionnaire?

### Critique by Cochrane Healthcare Quality Research Group, Cardiff University

Members of Cardiff University’s Cochrane Healthcare Quality Research group (http://www.hqcardiff.com/) participated in an iterative process to critique the developing conceptual map. The group comprised (in 2013) 10–15 healthcare quality researchers, including PhD students, clinician researchers and professional researchers. Available members of the group (10–15 people/meeting) attended three 60-min meetings at which a brief overview of the research was presented, including source data, methods of analysis and the developing conceptual map. Group members were asked to comment on the content of the conceptual map (concepts) and the graphical representation. Encounters were audio-taped with the consent of the group and key suggestions for improving the conceptual map were extracted and used to contribute further to development of the final conceptual map.

## Results

**Figure 2:**
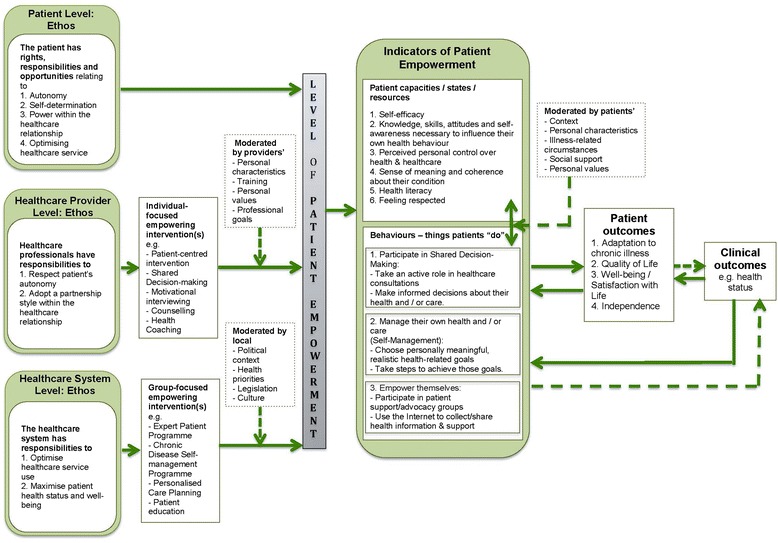
A conceptual model of patient empowerment

A brief summary of the findings from each data source will be presented, followed by presentation of the emergent conceptual map of patient empowerment (See Fig. 2). Analysis and synthesis across the two data sources enabled identification of five key components of patient empowerment: underpinning ethos, moderators, interventions, indicators and outcomes.

### Synthesis of the literature

A total of 164 citations were retrieved, and 20 articles were removed after title scanning. 144 abstracts were read and, after assessment, 108 full articles were subjected to detailed assessment. Forty-one of these (38 %) were removed because they did not include a definition of patient empowerment. 67 articles were finally included for full data extraction and analysis (See Additional file 2).

Overall, 52 % of the articles were classified as empirical research (16 used quantitative methods, 13 used qualitative methods, and six used mixed methods), 48 % as literature reviews, essays, commentaries and editorials. Empirical studies were conducted in Europe (68 %), North America (17 %) and Asia (15 %). Citations to 43 other published definitions of patient empowerment were identified among the 67 included articles, but 38 % of included articles did not cite a published definition of patient empowerment. Funnell et al’s [29, 30] definition of patient empowerment was the most frequently cited definition, cited by 11 % of the 67 included articles, followed by Aujoulat et al.[31] (6 %), Anderson et al. [5, 32, 33] (6 %), Lau [34] (5 %) and Gibson [35] (5 %). Table 3 provides a list of the most frequently cited definitions of patient empowerment identified (at least 3 citations or 5 %).

**Table 3:** Most frequently cited definitions of patient empowerment

Author	Definition	Cited by %
Funnell et al.	“We have defined the process of empowerment as the discovery and development of one’s inherent capacity to be responsible for one’s own life. People are empowered when they have sufficient knowledge to make rationale decisions, sufficient control and resources to implement their decisions, and sufficient experience to evaluate the effectiveness of their decisions. Empowerment is more than an intervention or strategy to help people make behaviour changes to adhere to a treatment plan. Fundamentally, patient empowerment is an outcome. Patients are empowered when they have knowledge, skills, attitudes, and self-awareness necessary to influence their own behaviour and that of others in order to improve the quality of their lives”(Funnell et al., 1991). “Empowerment is a patient-centered, collaborative approach tailored to match the fundamental realities of diabetes care. Patient empowerment is defined as helping patients discover and develop the inherent capacity to be responsible for one's own life” (Funnell & Anderson, 2004)	11 %
Aujoulat et al.	“Empowerment may be defined as a complex experience of personal change. It is guided by the principle of self-determination and may be facilitated by health-care providers if they adopt a patient-centred approach of care which acknowledges the patients’ experience, priorities and fears. In order to be empowering for the patient, therapeutic education activities need to be based on self-reflection, experimentation, and negotiation so as to allow for the appropriation of medical knowledge and the reinforcement of psychosocial skills”. (Aujoulat, d’Hoore, & Deccache, 2007)	6 %
Anderson et al.	“… The empowerment approach as a method for helping patients select and make changes in their diabetes self-management. This approach is based on the principles of counselling, and educational psychology, nursing and behavioural theory, and the reality of day-to-day management of a chronic disease such as diabetes”(Anderson & Funnell, 2002). “The empowerment process is regarded as an individual’s discovery (and development) of their inborn capacity to control and take responsibility for their live”(Anderson & Funnell, 2005). “Patient empowerment is a process designed to facilitate self-directed behavior change…The empowerment approach involves facilitating and supporting patients to reflect on their experience of living with diabetes. Self-reflection occurring in a relationship characterized by psychological safety, warmth, collaboration, and respect is essential for laying the foundation for self-directed positive change in behavior, emotions, and/or attitudes”(Anderson & Funnell, 2010)	6 %
Lau	“Patient empowerment in the health care context means to promote autonomous self-regulation so that the individual’s potential for health and wellness is maximised. Patient empowerment begins with information and education and includes seeking out information about one’s own illness or condition, and actively participating in treatment decisions”(Lau, 2002)	5 %
Gibson	“Empowerment is a social process of recognizing, promoting and enhancing people’s abilities to meet their own needs, solve their own problems and mobilize the necessary resources in order to feel in control of their own lives. Even more simply defined, empowerment is a process of helping people to assert control over the factors which affect their health”(Gibson, 1991)	5 %

Definitions of patient empowerment identified in the literature were diverse, with some definitions focusing on patient empowerment as a transformative process that patients go through as they gain control of their health and healthcare and adapt to having a chronic disease (LTC), others focusing on principles or ethos underpinning patient empowerment (e.g., autonomy, self-determination), and others defining patient empowerment in terms of interventions that aim to foster self-management of LTCs (Tables 3 and 4). Definitions of patient empowerment were also diverse in terms of whether they focused on the patient level, the healthcare provider level or the healthcare system level (Tables 3 and 4). The components identified in the qualitative analysis of these definitions are mapped in a diagram in Fig. 2 and described in more detail below.

**Table 4:** Exemplars demonstrating contribution of the three data sources to development of the conceptual map of patient empowerment

	Patient level	Health professional level	Healthcare system level
Ethos	*“Empowerment is based on the tenets of self-determination theory, which states that individuals are naturally motivated to improve their own well being”. (McCarley 2009 - Essay)* (See Additional file 1)	*“Empowerment is also about respecting patients’ rights and voice” (The Lancet 2012 - Essay)* (See Additional file 1)	*“Promoting autonomous self-regulation so that the individual's potential for health and wellness is maximized” (Quantin 2011 – Review study)* (See Additional file 1)
	*“[Patient empowerment] philosophy is based on the assumption that to be healthy, people must be able to bring about changes, not only in their personal behaviour, but also in their social situations and the organisations that influence their lives” (Holmstrom 2010 – Review study)* (See Additional file 1)	*“[Patient empowerment] changes the balance within the doctor–patient relationship, making it more democratic in the sense that power is more equally distributed. (Batifoulier 2011 - Essay)* (See Additional file 1)	*“[Patient empowerment is] based on the assumption that people require psychosocial skills to bring about changes in their personal behaviour, their social situations, and the institutions that influence their lives”. (Lo 2012 - Editorial)* (See Additional file 1)
	*“Self-responsibility for health… understanding that me, myself and I is an important participant in my issues and my health” (Health manager - Interview)*	*“You put a person with a lung condition in front of a doctor or a nurse and they become a patient… if you really want true empowerment you gave back a bit of the control to the patient” (Patient representative - Interview)*	*“Patient empowerment is considered philosophy of health care that proceeds from the perspective that optimal outcomes of health care interventions are achieved when patients become active participants in the health care process”. (Bos 2008 - Essay)* (See Additional file 1)
	*“The patients’ role is changing from a patronized patient to an informed patient and further to a responsible, autonomous and competent partner in his or her own care”. (Ammenwerth 2011 – Review study)* (See Additional file 1)	*“More active role in consultations and health decision making and moving away from the traditional asymmetric power balance inherent in the medical model”. (Bartlett 2011 – Quantitative study)* (See Additional file 1)	*“I think as we said it’s being satisfied with the service but also on the occasions when you need to be able to make a choice that you have choices available to you within reason” (Patient - interview)*
	*“I just like the idea of being a person who has opinions and is listened to especially when it concerns me” (Patient - interview)*		
	*“Empowerment is the authority, the right, yes, your right to be able to take decisions and do for yourself.” (Patient - interview)*		
Moderators	*“Even the most empowered people that I know when going to seek healthcare particularly if something is really seriously wrong with them they’re not interested in making a shared decision” (Health researcher - Interview) [disease characteristics]* *“A lot of patients actually go into consultations with a fair bit of knowledge around what their options might be to start with and then really very much down to the clinician’s personality”. (Health researcher - Interview) [health literacy]*	*“Empowerment means to me that they (patients) are given up-to-date, clinically based evidence and treatment the patient is given by trained practitioners”. (Clinician - interview)* *“Health providers will promote empowerment as long as this is in line with their goals, but they will also need training so they know what to do and how to do it” (Experts group)*	*“Whether or not an organisation is person focused, whether they put the customer right at the centre of the organisation” (Health researcher - Interview)* *“I guess it is about what it is important to the government at the time, so if there is an interest in patient empowerment, then people are more likely to access empowering interventions” (Experts group)*
Interventions		*“The capacity-building process whereby individuals increase their belief that they play an active role in their care (ie, taking action to solve their problems)”. (Alegria 2008 – Qualitative study)* (See Additional file 1)	*“A social process of recognising, promoting and enhancing peoples’ abilities to meet their own needs, solve their own problems and mobilise the necessary resources in order to be in control of their lives”. (Hiley 2008 – Mixed methods study)* (See Additional file 1)
		*“Motivational interviewing well as a health professional you are trying to get a sense of what somebody’s about and help them to understand what they are about themselves so that that can be taken into consideration”. (Clinician - Interview)*	*“Another facet to patient empowerment which has been tried in numerous general practices in the formation of patient participation groups. And that’s um really left as a clinical empowerment but more geared towards getting patients to have a say in the running of a General Practice”. (Health manager - Interview)*
		*“The relationship issues can be part of empowerment so you go to see the GP and the GP is kind of supportive and positive and doesn’t undermine the patient’s efforts then the patient may come out feeling empowered” (Health researcher - interview)*	*“As for health system interventions, we should consider the Expert Patient Programme, and what people are achieving through that in terms of empowerment” (Experts group)*
		*You’re giving them the wherewithal, the tools necessary to take rational decisions, to understand and to act upon advice” (Patient - interview)*	
	Things patients do	Patients’ capacities	Outcomes
Indicators and Outcomes	*“Empowering patients can enable them to take more responsibility for managing their health and encourage self-management activities”. (Alpay 2010 – Review study)* (See Additional file 1)	*“Patients […] manage their own condition and feel like they have got the ability and are given confidence to be able to manage their condition” (Health provider - interview)*	*“WHO has described empowerment as a “prerequisite for health” and “a proactive partnership and patient self-care strategy to improve health outcomes and quality of life among the chronically ill” (Ayme 2008 – Review study)* (See Additional file 1)
	*“Empowerment reflects a type of support that enables and motivates people to take the necessary steps to manage and improve their health in a self-directed manner”. (Bann 2010 – Mixed methods study)* (See Additional file 1)	*“People obtaining the knowledge and skills to make it possible for them to become active partners, with professionals, in making informed decisions and choices about their own treatment and care”. (Boudioni 2012 – Quantitative study)* (See Additional file 1)	*“Process of change in which patients positively reach a new perspective by reconceptualising and reinterpreting their disease”. (Hagiwara 2011 – Review study)* (See Additional file 1)
	*“Patients […] manage their own condition and feel like they have got the ability and are given confidence to be able to manage their condition” (Health provider - interview)*	*“Helping people to discover and use their own innate ability to gain mastery over their disease or status”. (Topac 2011 - Essay* (See Additional file 1)*)*	*“It’s very important for doctors to empower patients to make sure they’ve got information, make sure that they’ve got strategies for dealing with their condition so it minimises the impact of it on their quality of life”. (Patient representative - interview)*
	*“More active role in consultations and health decision making and moving away from the traditional asymmetric power balance inherent in the medical model”. (Bartlett 2011 – Quantitative study)* (See Additional file 1)	*“[Patient empowerment is] an individual trait, characterized by an emphasis on increased individual control over the different aspects of one’s life” (Oh 2012 – Quantitative study)* (See Additional file 1)	
	*“[Empowerment is] a process of personal transformation”. (Falcao-Reis 2010 - Essay)* [43]	*“The capacity-building process whereby individuals increase their belief that they play an active role in their care (ie, taking action to solve their problems)”. (Alegria 2008 – Qualitative study)* (See Additional file 1)	
	*“An individual’s discovery (and development) of their inborn capacity to control and take responsibility for their lives”. (Petersen 2008 - Essay)* (See Additional file 1)	*[patients] have expertise in how their condition affect them and I think medical professionals it’s vital that they acknowledge that expertise […] part of patient empowerment is the medical professional respecting that expertise and trying to use that and draw on that as a resource… I think the other part of it is the medical professional using their skills and their knowledge um to help the patient learn new strategies, new techniques and develop their own knowledge so that they’re better equipped to care for themselves. (Patient representative - interview)*	
	*“I think it’s just really useful to talk and share, share your experiences with other people and then you listen to them”. (Patient - interview)*	*… it’s a matter of whether the interaction with the person or system you just had um not just identifies the problem […] you’ve got […] gives you treatment but makes you feel more confident, capable of […] dealing with it…. (Patient - interview)*	
	*“It’s not people telling me what I need. It’s me telling them what I need” (Patient - interview)*	*… encouraging patients to um attain a greater health literacy so that they can manage their chronic disease better themselves. That they know when to seek help, they know how to self-manage and that will improve their outcomes. […] knowing what services to access and when. So a better self-reliance in terms of managing minor illness … (Patient - interview)*	
	*You’re giving them the wherewithal, the tools necessary to take rational decisions, to understand and to act upon advice” (Patient - interview)*	*… having sufficient information to give you confidence to make a decision to the best of your abilities whatever those are and those obviously will vary from person to person… People who understand their lung condition a bit better are often the ones who are confident to actually make some choices themselves in a more able way. (Patient representative - interview)*	

Thematic analysis of the published definitions of patient empowerment enabled development of an initial conceptual map of patient empowerment to be drafted, identifying key underpinning assumptions (later changed to underpinning ethos), interventions, moderators, indicators and outcomes of patient empowerment. Table 4 presents the five emerging components in narrative form with exemplars illustrating how each of the data sources, and critique by the Cochrane Healthcare Quality Research group contributed.

### Interviews with key informants

Nineteen interviews were conducted: four with primary care clinicians, four with health researchers, four with health managers, four with patients affected by LTCs, and three with patient representatives from organisations supporting people affected by LTCs. Most participants were female (62 %). Interviews lasted on average 27 min. The shortest interview was carried out with a health manager and lasted 15.51 min. The longest interview was given by a patient representative and lasted 54.36 min. Although interviews were intended to last approx. 45 min, interviews were not rushed and interview duration was driven by topic discussion and willingness of participants to contribute. The initial (interim) framework developed from thematic analysis of the definitions of patient empowerment extracted in the scoping literature review guided analysis of the interview transcripts.

The interviews contributed further to identification of underpinning ethos, interventions, moderators, indicators and outcomes of patient empowerment. Exemplars illustrating contributions of the interviews to development of the conceptual map in narrative form are presented in Table 4. When asked about patient outcomes, many participants mentioned that although empowerment is a key benefit for people with LTCs, they found it difficult to define or explain what they understood patient empowerment to be. This is illustrated in the following extract from an interview with a Patient representative affected by a LTC:

*“If you went to a group of people and said ‘how can we empower you?’ I think they might look at you as if you were a bit mad really because I think even most intelligent group of patients would wonder what the definition of that is. It’s interesting because you started off asking me what I thought empowerment was almost so I think there’s in my experience there’s lots of different definitions to that”.*

This supports findings from the scoping literature review regarding diversity of patient empowerment definitions, and lack of clarity and consensus on what patient empowerment means. Although data saturation was not confirmed in the present study, it was notable that some healthcare providers emphasised patient responsibility for LTC self-management, whilst patients were more inclined to emphasise control, as demonstrated by these two contrasting quotes:

“Self-responsibility for health… understanding that me, myself and I is an important participant in my issues and my health” *(Health manager)*

“You put a person with a lung condition in front of a doctor or a nurse and they become a patient… if you really want true empowerment you gave back a bit of the control to the patient” *(Patient representative)*

Regular critique of the developing conceptual map by the Cochrane Healthcare Quality Research group helped to further clarify underpinning ethos, interventions, moderators, indicators and outcomes of patient empowerment. Some of the key suggestions made by group members are included in Table 4 (attributed to Experts group). Importantly, these discussions contributed to developing relationships described among components of the conceptual map, and facilitated graphical representation. At the final meeting, the group suggested (i) changing the wording in the “Healthcare System Level: Ethos” box from *Minimise healthcare service use* to *Optimise healthcare service use*, and to add *Optimise healthcare service use* to the “Patient Level: Ethos” box, as well as the “Healthcare Provider Level: Ethos” box and (ii) removing *Optimism/hope* from the “Indicators of Patient Empowerment”/“Patient capacities/states/resources” box, because patient optimism and hopefulness may be unrealistic as healthcare outcome goals. The group commented that the importance of patients feeling positive was already included under “perceived personal control”/“self-efficacy”, and under “well-being” (Patient outcomes).

#### Synthesis: components of the emergent conceptual map of patient empowerment

Findings from the two sources were synthesised to produce a graphical representation, shown in Fig. 2, illustrating emergent model components (underpinning ethos, interventions, moderators, indicators and outcomes of patient empowerment) and emergent relationships between components. Table 4 provides examples of how the data sources contributed to identifying components of the emergent conceptual map.

##### Underpinning ethos

Underpinning ethos (principles or values) were identified in the qualitative thematic analysis of published definitions of patient empowerment, and were supported by and further developed using the qualitative interview data. These were identified at three levels: the patient level, the healthcare provider level and the healthcare system level:

1. Patient level ethos: The patient has rights, responsibilities and opportunities relating to autonomy, self-determination and power within the healthcare relationship, as well as to optimise healthcare service use.
2. Healthcare provider level ethos: Healthcare providers have responsibilities to respect patient autonomy and adopt a partnership style within the healthcare relationship.
3. Healthcare system level ethos: The health system seeks to support patients with long-term conditions to self-manage their condition so they can optimise healthcare service use and maximise patient health status and well-being.

##### Empowering interventions

Analysis of published definitions of patient empowerment, and analysis of UK stakeholder interviews suggested that the level of patient empowerment is modifiable by healthcare interventions that can be implemented by healthcare providers or healthcare systems to promote patient empowerment. Examples of healthcare provider-level interventions include patient centred training interventions [36], shared decision-making [37], motivational interviewing [38], counselling, health coaching, and signposting to support services. Examples of healthcare-system level interventions include training programmes for clinicians and/or patients, with or without supporting educational materials [36] e.g., the Expert Patient Programme, the Chronic Disease Self-Management Programme [39, 40], Personalised Care Planning, and patient education programmes.

##### Moderators of patient empowerment

Several moderator variables that could influence patient empowerment were identified at the patient, healthcare provider and health system levels. These are variables that may influence how effectively the empowering interventions influence patient empowerment. So, for example, in the case of healthcare providers implementing a shared decision-making intervention, the impact of this on patient empowerment is influenced by variables such as the healthcare provider’s personal characteristics, training, personal values and professional goals. At the patient level, the patient’s ability to undertake patient empowerment activities can be influenced by variables such as the patient’s context, personal characteristics, values, social support as well as by the circumstances of their disease (e.g., duration, severity). At the healthcare system level, the political context, legislation, health priorities and culture were identified as moderator variables that could influence how patient empowerment initiatives are implemented by the healthcare system.

##### Indicators of patient empowerment

Analysis identified that patient empowerment can be conceived of as a patient “state” and by patient behaviours. Patients can be placed somewhere on a spectrum from lower to higher levels of the variable “empowerment”. Empowered patients “*feel like they have got the ability and are given confidence to be able to manage their condition” (Healthcare provider - interview).* This “state” can be indicated by

1. Patient capacities, beliefs or resources including self-efficacy, sense of meaning and coherence about their condition, health literacy, perceived control and feelings respected by their healthcare providers
2. Activities or behaviours (things patient *do*) e.g., participate in shared decision-making by taking an active role and making informed decisions about their health and healthcare, self-manage their condition by choosing meaningful and realistic goals and taking steps to achieve those goals, participate in collective activities such as patient support or advocacy groups, and search for information about their health condition e.g., on the internet.

Data analysis suggested the (as yet untested) hypotheses that

- Hypothesis 1: Empowered patients will report higher levels of self-efficacy, sense of meaning and coherence about their condition, health literacy, perceived control and feeling respected by their healthcare providers.

Patient empowerment is, therefore, a variable that could be operationalised in a patient-reported measure.

- Hypothesis 2: Empowered patients, those scoring high on indicators of the variable “state” patient empowerment, will have better self-reported outcomes e.g., (a) be better adapted to their condition; (b) have improved quality of life; (c) report higher levels of well-being and satisfaction with life; (d) achieve some independence relating to their healthcare.

These outcomes are also variables that could be operationalised in (a) patient-reported measure(s).

- Hypothesis 3: There is a dual reciprocal relationship between patient empowerment activities or behaviours (things patient *do*) and patient empowerment capacities, beliefs or resources. For example, patients who use the internet to collect health information may have improved health literacy and patients with high levels of health literacy may make more informed decisions about their health.

##### Outcomes of patient empowerment

Data suggested that patient empowerment is likely to lead to better patient outcomes such as better adaptation to their LTC, better quality of life and well-being, and more independence from healthcare providers and carers. One patient said:

“It’s very important for doctors to empower patients to make sure they’ve got information, make sure that they’ve got strategies for dealing with their condition so it minimises the impact of it on their quality of life”. *(Patient representative - interview)*

Improved clinical outcomes e.g., health status may be more tentative, distal and long-term outcomes of patient empowerment, as demonstrated by the dashed line between the “patient outcomes” and “clinical outcomes” and between “indicators of patient empowerment” and “clinical outcomes” boxes in Fig. 2. Improved health status was described in some areas of the literature as an outcome of patient empowerment initiatives e.g., self-management training (e.g., Camerini et al., 2012; Chang 2012; Moattari 2012; see Additional file 2). However, clinical outcomes could themselves have an influence on patient outcomes and on empowerment, suggesting a fourth hypothesis:

- Hypothesis 4: There are dual reciprocal causal relationships between patient empowerment indicators, patient outcomes and clinical outcomes.

For example, patients whose health is deteriorating as a result of a degenerative LTC may have reduced independence, quality of life and levels of life satisfaction, with consequent reduction in feelings of self-efficacy and perceived control over their health, and may become less able to manage their own health. Some patients may feel empowered, and report high levels of self-efficacy, knowledge, perceived control and they may also feel respected by their healthcare providers, yet at the same time, they may lack functional and critical health literacy. In other words, patients may feel they know enough about their condition, yet their knowledge may be incorrect, and so self-management activities they engage in may put at risk achievement of the positive outcomes that they hope for or expect. However, other patient empowerment capacities e.g., self-efficacy, when *interacting* with patient empowerment activities such as participating in shared decision-making and patient support and advocacy groups, as well as using the internet to collect health information may be more likely to increase health literacy and contribute to achieving positive patient outcomes. This is illustrated in Fig. 2.

## Discussion

The conceptual map presented in this study is a novel contribution to the literature, as it maps five key components of patient empowerment identified in published definitions of patient empowerment and/or in interviews with 19 UK stakeholders. These components are: underpinning ethos, interventions, moderators, indicators and outcomes of patient empowerment. The conceptual map includes relationships with other constructs such as health literacy, self-management and shared decision-making.

Findings are consistent with the principles and approaches of patient-centered care, as the conceptual map describes that healthcare providers adopt a partnership style with patients, and provide healthcare that is respectful of patients to support informed patient decision-making. Considering the lack of consensus identified in this study about what patient empowerment means to clinicians, patients and researchers [41, 42], the conceptual map presented here contributes to a clearer understanding of patient empowerment for LTCs.

However, this study has some methodological limitations. The scoping literature review included only articles that had the words “patient” and “empowerment” in the title or abstract, and may therefore have excluded some published definitions of patient empowerment. The qualitative interviews included only a small sample of key stakeholders, and interview findings are not generalizable beyond the sample recruited in this study. Funding and time constraints meant that data saturation could not be confirmed. Furthermore, interviews were conducted with UK stakeholders only, and the views of people working/accessing care outside the UK were not included. This may limit applicability of the findings. However this geographical limitation is partly mitigated by inclusion of studies from Asia, North America and Europe in the scoping literature review. It will be important to validate these findings with larger samples of each of these stakeholder groups within and beyond the UK, and this could lead to further development of the conceptual map.

Indicators of patient empowerment identified in this study could be interpreted within the model proposed by de Haes & Benzing (2009) that distinguishes immediate, intermediate and long-term outcomes from medical communication [43]. The activities/behaviours (things patient *do*) e.g., participate in shared decision-making could be considered immediate outcomes of patient empowerment. The patient outcomes e.g., quality of life and well-being could be considered intermediate outcomes of patient empowerment, with health status as a possible long term outcome. These immediate, intermediate and long term outcomes of patient empowerment may be useful to operationalise patient empowerment, and test these hypotheses.

Health literacy is included as one indicator of patient empowerment because patients need to be able to understand medical information in order to use it effectively to contribute to shared decision-making and to manage their own LTC. Nutbeam’s model of health literacy includes functional, interactive and critical aspects, with all three dimensions proposed to influence the effectiveness with which patients make use of health information [44]. This is supported by evidence that health literacy may influence how effectively information is communicated between patient and healthcare provider in shared decision making [45, 46] and these authors have argued that patients may indeed be disempowered by consultations in which they cannot understand the medical information communicated with them. However, patients with LTCs can also empower themselves by seeking health information on the internet that may have a positive impact on their capacity to communicate effectively with healthcare providers and contribute to shared decision-making. This is supported by the recent assertions that (a) health literacy and patient empowerment are distinct concepts [47], and (b) that health literacy is necessary but insufficient to ensure patient empowerment [43].

Shared decision-making is described as an intervention at the healthcare provider level that may promote patient empowerment. This is consistent with published models of shared decision making e.g., Elwyn et al.’s 2012 three-step model proposing that shared decision making can be implemented by individual healthcare professionals by (i) introducing a choice to the patient, then (ii) describing the options (sometimes supported by decision support tools), and (iii) helping patients to explore their preferences and then make a decision [48]. Patient self-management is described to be an activity that is undertaken by empowered patients, by choosing personally meaningful, realistic health related goals, and taking steps to achieve those goals. This is consistent with models of LTC self-management that advocate sharing of knowledge and authority between patients and healthcare providers, and that promote LTC-related problem solving such as the Arthritis Self-Management programme [49].

Three sets of ethos underpinning published patient empowerment definitions were identified, each focusing on responsibilities and/or opportunities at the patient level, the healthcare provider level or the healthcare system level. These ethos support published philosophical principles relating to patient empowerment, such as patient autonomy and self-determination [50–52], whilst also identifying that patients also have responsibilities to optimise their own healthcare service use. Patient responsibilities in LTC self-management have also been identified by others, for example Holman & Lorig (2004) argued that patients with LTCs have responsibilities to use medications properly, change their health behaviour to improve symptoms or slow disease progression, adjust to the social and economic consequences of their condition, cope with the emotional consequences of their condition, and report symptoms accurately to their healthcare provider [49].

When operationalizing patient empowerment e.g., in clinical practice, it may be helpful to consider that relevant actors (patients, healthcare providers and the healthcare system) may be constrained by some of the moderators identified in this study and that some interventions may be better targeted at the patient level, some at the healthcare provider level, and others at the healthcare system level. For example, the capacity of individual healthcare providers to empower patients may be limited by their training, personal values and professional goals. Training interventions to support healthcare providers to implement shared decision-making may therefore be effective. The capacity of patients to empower themselves may be limited by their personal characteristics, their access to social support, and their personal values. Healthcare providers could consider encouraging patients to identify sources in their social network who could support them to choose personally meaningful, realistic health-related goals and take steps to achieve those goals.

A variety of interventions may promote levels of individual patient empowerment. Some of these may best be targeted at patients themselves, such as interventions aimed at improving patients’ use of the internet for health information [53]. Others may be most usefully targeted at clinicians (e.g., skills training in motivational interviewing or counselling), and yet others may best be targeted at the healthcare system level (e.g., Expert Patient Programme).

A range of indicators, such as patient self-efficacy, perceived personal control, and participation in shared decision-making and self-management activities may be useful to assess levels of individual patient empowerment. These may be useful in evaluating interventions that aim to promote patient empowerment. Measures of some of these indicators have already been used to evaluate interventions that aim to promote patient empowerment. For example, measures of self-efficacy have been used to evaluate outcomes from the UK Expert Patient Programme, and other patient-led education and coaching interventions [9, 54]. Empowered patients may be better adapted to their health condition, more independent, with better quality of life and well-being and this could have an impact on more distal clinical outcomes such as health status, although this remains to be consistently demonstrated.

However, to date there is no evidence of a single measure that can adequately capture all the outcomes (indicators) of patient empowerment identified in this study [1, 2]. Some efforts have been made to develop generic measures of patient empowerment [1, 2, 55], a recent example being a measure published by Small et al. in 2013 [56]. But it is not clear whether definitions used to develop existing measures of patient empowerment reflect a widely accepted construct of patient empowerment.

Next steps in research could include, in addition to validating the conceptual map with larger samples and in other contexts, seeking broader consensus amongst international patients, clinicians and health researchers about the content of the conceptual map to achieve a shared understanding of patient empowerment. This could result in further development of the conceptual map. It may also be useful to identify and assess available instruments that purport to capture patient empowerment to determine whether a single instrument can capture all the indicators of patient empowerment described in this study. It would also be helpful to review and assess available measures that capture the individual patient empowerment indicators identified here e.g., measures of illness identity (sense of meaning and coherence), self-efficacy and perceived personal control. Together, these approaches could help to identify the best approach to measuring patient empowerment, and to design robust research to evaluate interventions that aim to promote patient empowerment.

## Conclusion

This article presents a novel conceptual map of patient empowerment grounded in published definitions of patient empowerment and qualitative interviews with UK stakeholders. The value of this work is in bringing some order to the diverse field of patient empowerment. The conceptual map may be useful to clinicians, health managers and researchers who are designing, implementing and evaluating interventions that aim to promote patient empowerment.

## Additional files

Additional file 1:
**RATS Checklist.**
Additional file 2:
**Details of included articles.**

## Abbreviations

CINAHL: Cumulative Index to Nursing & Allied Health Literature
EMBASE: Excerpta Medica dataBASE
EU: European Union
LTC: Long term condition
NHS: National Health Service
PROM: Patient-reported outcome measure
PsycInfo: Indexing database produced by the American Psychological Association
UK: United Kingdom of Great Britain and Northern Ireland
