# Supplementary material for: Conceptualising patient empowerment: a mixed methods study
Source: BMC Health Serv Res. 2015 Jul 1;15:252. doi: 10.1186/s12913-015-0907-z (PMC4488113; doi:10.1186/s12913-015-0907-z)
Supplement: Additional file 1: — RATS Checklist. [file 12913_2015_907_MOESM1_ESM.docx]

**Conceptualising patient empowerment: a mixed methods study**

Bravo P, Edwards A, Barr PB, Scholl I, Elwyn G, McAllister M.

RATS Checklist

|  | **R Relevance of study question** |  |
| --- | --- | --- |
| 1  2 | Research question explicitly stated  Research question justified and linked to the existing knowledge base (empirical research and policy) - YES | [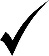](https://www.google.co.uk/imgres?imgurl=http://www.clipartbest.com/cliparts/jRT/Gnk/jRTGnkEiL.png&imgrefurl=http://www.clipartbest.com/check-symbol-in-powerpoint&docid=68WbSLWcx9MDwM&tbnid=dJLbrcnNUAc39M:&w=437&h=500&ei=YuJPU5m4BImy0QWDnoCICg&ved=0CAIQxiAwAA&iact=c)  [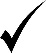](https://www.google.co.uk/imgres?imgurl=http://www.clipartbest.com/cliparts/jRT/Gnk/jRTGnkEiL.png&imgrefurl=http://www.clipartbest.com/check-symbol-in-powerpoint&docid=68WbSLWcx9MDwM&tbnid=dJLbrcnNUAc39M:&w=437&h=500&ei=YuJPU5m4BImy0QWDnoCICg&ved=0CAIQxiAwAA&iact=c) |
|  | **A Appropriateness of qualitative method** |  |
| 3 | Study design described and justified i.e., why was a particular method (e.g., interviews) chosen? YES | [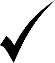](https://www.google.co.uk/imgres?imgurl=http://www.clipartbest.com/cliparts/jRT/Gnk/jRTGnkEiL.png&imgrefurl=http://www.clipartbest.com/check-symbol-in-powerpoint&docid=68WbSLWcx9MDwM&tbnid=dJLbrcnNUAc39M:&w=437&h=500&ei=YuJPU5m4BImy0QWDnoCICg&ved=0CAIQxiAwAA&iact=c) |
|  | **T Transparency of procedures** |  |
|  | Sampling: |  |
| 4 | Criteria for selecting the study sample justified and explained? YES: purposive to achieve diversity of opinion | [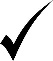](https://www.google.co.uk/imgres?imgurl=http://www.clipartbest.com/cliparts/jRT/Gnk/jRTGnkEiL.png&imgrefurl=http://www.clipartbest.com/check-symbol-in-powerpoint&docid=68WbSLWcx9MDwM&tbnid=dJLbrcnNUAc39M:&w=437&h=500&ei=YuJPU5m4BImy0QWDnoCICg&ved=0CAIQxiAwAA&iact=c) |
|  | Recruitment: |  |
| 5 | Details of how recruitment was conducted and by whom: YES –Clinicians, health managers and academic health researchers were identified through professional networks and contact information on institutional websites and were contacted directly by PBr by email or letter and sent a letter of invitation to participate in one face-to-face or telephone interview. Patients and patient representatives were identified through patient support organisations for LTCs in Wales. Key members within the organisations received a letter in which the study was described and were asked to recruit patient and patient representative members of their organisation to take part in the study. Information sheets were provided and participants were asked to return a reply slip to PBr should they wish to participate in the study | [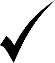](https://www.google.co.uk/imgres?imgurl=http://www.clipartbest.com/cliparts/jRT/Gnk/jRTGnkEiL.png&imgrefurl=http://www.clipartbest.com/check-symbol-in-powerpoint&docid=68WbSLWcx9MDwM&tbnid=dJLbrcnNUAc39M:&w=437&h=500&ei=YuJPU5m4BImy0QWDnoCICg&ved=0CAIQxiAwAA&iact=c) |
| 6 | Details of who chose not to participate and why: YES – Thirty-four potential participants were approached (10 patients and patient representatives, 6 clinicians, 10 health managers, and 8 health researchers). Non participants (15 out of 34) were unable to arrange time for the interview because they were too busy. | [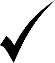](https://www.google.co.uk/imgres?imgurl=http://www.clipartbest.com/cliparts/jRT/Gnk/jRTGnkEiL.png&imgrefurl=http://www.clipartbest.com/check-symbol-in-powerpoint&docid=68WbSLWcx9MDwM&tbnid=dJLbrcnNUAc39M:&w=437&h=500&ei=YuJPU5m4BImy0QWDnoCICg&ved=0CAIQxiAwAA&iact=c) |
|  | Data collection: |  |
| 7 | Method(s) outlined and examples given (e.g., interview questions) – YES, See Tables 1 & 2 | [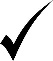](https://www.google.co.uk/imgres?imgurl=http://www.clipartbest.com/cliparts/jRT/Gnk/jRTGnkEiL.png&imgrefurl=http://www.clipartbest.com/check-symbol-in-powerpoint&docid=68WbSLWcx9MDwM&tbnid=dJLbrcnNUAc39M:&w=437&h=500&ei=YuJPU5m4BImy0QWDnoCICg&ved=0CAIQxiAwAA&iact=c) |
| 8 | Study group and setting clearly described - YES | [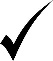](https://www.google.co.uk/imgres?imgurl=http://www.clipartbest.com/cliparts/jRT/Gnk/jRTGnkEiL.png&imgrefurl=http://www.clipartbest.com/check-symbol-in-powerpoint&docid=68WbSLWcx9MDwM&tbnid=dJLbrcnNUAc39M:&w=437&h=500&ei=YuJPU5m4BImy0QWDnoCICg&ved=0CAIQxiAwAA&iact=c) |
| 9 | End of data collection justified and described. YES - Data collection was complete when data saturation was reached, with no new themes emerging in interviews. | [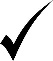](https://www.google.co.uk/imgres?imgurl=http://www.clipartbest.com/cliparts/jRT/Gnk/jRTGnkEiL.png&imgrefurl=http://www.clipartbest.com/check-symbol-in-powerpoint&docid=68WbSLWcx9MDwM&tbnid=dJLbrcnNUAc39M:&w=437&h=500&ei=YuJPU5m4BImy0QWDnoCICg&ved=0CAIQxiAwAA&iact=c) |
|  | Role of researchers: |  |
| 10 | Do the researchers occupy dual roles (clinician and researcher)? Are the ethics of this discussed? YES, AE, IS and MM were working as both clinicians and researchers at the time of the study. However, the ethics of this are not discussed in the manuscript because no ethical issues were raised by these circumstances in the study. PBr, who collected the data, was not working in a clinical role during the study period, and MM, AE and IS had no clinical relationship with any of the participants recruited in the study. | 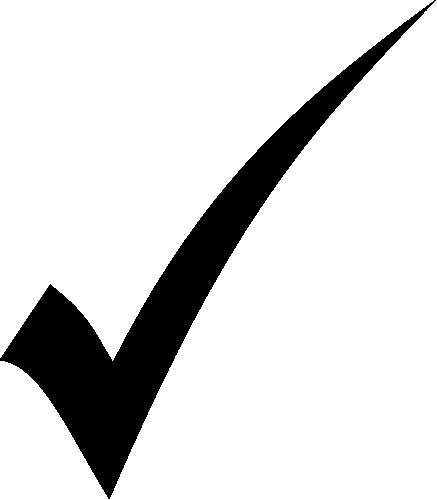 |
| 11 | Do the researcher(s) critically examine their own influence on the formulation of the research question, data collection, and interpretation? YES – this was a descriptive study only, which was about concepts and not about actual care; this is mentioned in the Discussion (para 2). Also, see item 10 above. | 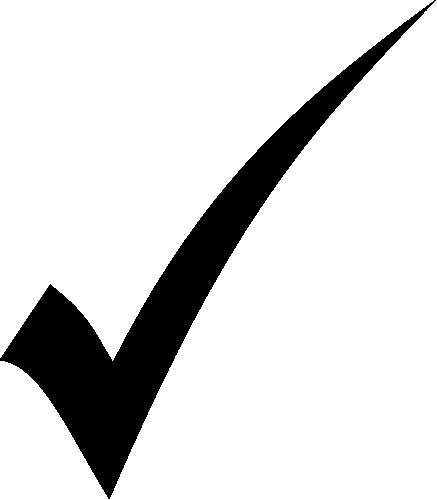 |
|  | Ethics: |  |
| 12 | Informed consent process explicitly and clearly detailed | [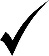](https://www.google.co.uk/imgres?imgurl=http://www.clipartbest.com/cliparts/jRT/Gnk/jRTGnkEiL.png&imgrefurl=http://www.clipartbest.com/check-symbol-in-powerpoint&docid=68WbSLWcx9MDwM&tbnid=dJLbrcnNUAc39M:&w=437&h=500&ei=YuJPU5m4BImy0QWDnoCICg&ved=0CAIQxiAwAA&iact=c) |
| 13 | Anonymity and confidentiality discussed | [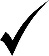](https://www.google.co.uk/imgres?imgurl=http://www.clipartbest.com/cliparts/jRT/Gnk/jRTGnkEiL.png&imgrefurl=http://www.clipartbest.com/check-symbol-in-powerpoint&docid=68WbSLWcx9MDwM&tbnid=dJLbrcnNUAc39M:&w=437&h=500&ei=YuJPU5m4BImy0QWDnoCICg&ved=0CAIQxiAwAA&iact=c) |
| 14 | Ethics approval cited | [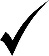](https://www.google.co.uk/imgres?imgurl=http://www.clipartbest.com/cliparts/jRT/Gnk/jRTGnkEiL.png&imgrefurl=http://www.clipartbest.com/check-symbol-in-powerpoint&docid=68WbSLWcx9MDwM&tbnid=dJLbrcnNUAc39M:&w=437&h=500&ei=YuJPU5m4BImy0QWDnoCICg&ved=0CAIQxiAwAA&iact=c) |
|  | **S Soundness of interpretive approach** |  |
| 15 | Analytic approach described in depth and justified? | [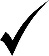](https://www.google.co.uk/imgres?imgurl=http://www.clipartbest.com/cliparts/jRT/Gnk/jRTGnkEiL.png&imgrefurl=http://www.clipartbest.com/check-symbol-in-powerpoint&docid=68WbSLWcx9MDwM&tbnid=dJLbrcnNUAc39M:&w=437&h=500&ei=YuJPU5m4BImy0QWDnoCICg&ved=0CAIQxiAwAA&iact=c) |
| 16 | *Indicators of quality:* Description of how themes were derived from the data (inductively) | [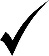](https://www.google.co.uk/imgres?imgurl=http://www.clipartbest.com/cliparts/jRT/Gnk/jRTGnkEiL.png&imgrefurl=http://www.clipartbest.com/check-symbol-in-powerpoint&docid=68WbSLWcx9MDwM&tbnid=dJLbrcnNUAc39M:&w=437&h=500&ei=YuJPU5m4BImy0QWDnoCICg&ved=0CAIQxiAwAA&iact=c) |
| 17 | Evidence of alternative explanations being sought? NO – the aim of the study was not to develop any explanations but rather to map out key themes underpinning published definitions of patient empowerment and to supplement this with the views of key stakeholders about what are the components of patient empowerment. | [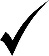](https://www.google.co.uk/imgres?imgurl=http://www.clipartbest.com/cliparts/jRT/Gnk/jRTGnkEiL.png&imgrefurl=http://www.clipartbest.com/check-symbol-in-powerpoint&docid=68WbSLWcx9MDwM&tbnid=dJLbrcnNUAc39M:&w=437&h=500&ei=YuJPU5m4BImy0QWDnoCICg&ved=0CAIQxiAwAA&iact=c) |
| 18 | Analysis and presentation of negative or deviant cases? NO – again because the aim of the study was not to develop an explanatory model but rather to map out key themes underpinning published definitions of patient empowerment and to supplement this with the views of key stakeholders about what are the components of patient empowerment, any analysis of negative or deviant cases that contradicted any emerging hypotheses was not relevant. This was a descriptive study only. | [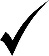](https://www.google.co.uk/imgres?imgurl=http://www.clipartbest.com/cliparts/jRT/Gnk/jRTGnkEiL.png&imgrefurl=http://www.clipartbest.com/check-symbol-in-powerpoint&docid=68WbSLWcx9MDwM&tbnid=dJLbrcnNUAc39M:&w=437&h=500&ei=YuJPU5m4BImy0QWDnoCICg&ved=0CAIQxiAwAA&iact=c) |
| 19 | Description of the basis on which quotes were chosen. YES - Table 4 makes it clear that quotations were selected as exemplars to demonstrate the contribution of the two data sources to development of the conceptual map of patient empowerment. | [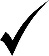](https://www.google.co.uk/imgres?imgurl=http://www.clipartbest.com/cliparts/jRT/Gnk/jRTGnkEiL.png&imgrefurl=http://www.clipartbest.com/check-symbol-in-powerpoint&docid=68WbSLWcx9MDwM&tbnid=dJLbrcnNUAc39M:&w=437&h=500&ei=YuJPU5m4BImy0QWDnoCICg&ved=0CAIQxiAwAA&iact=c) |
| 20 | Semi-quantification when appropriate – YES (See Table 3) | [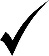](https://www.google.co.uk/imgres?imgurl=http://www.clipartbest.com/cliparts/jRT/Gnk/jRTGnkEiL.png&imgrefurl=http://www.clipartbest.com/check-symbol-in-powerpoint&docid=68WbSLWcx9MDwM&tbnid=dJLbrcnNUAc39M:&w=437&h=500&ei=YuJPU5m4BImy0QWDnoCICg&ved=0CAIQxiAwAA&iact=c) |
| 21 | Illumination of context and/or meaning, richly detailed – YES, to the extent that was relevant to the research question. | [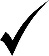](https://www.google.co.uk/imgres?imgurl=http://www.clipartbest.com/cliparts/jRT/Gnk/jRTGnkEiL.png&imgrefurl=http://www.clipartbest.com/check-symbol-in-powerpoint&docid=68WbSLWcx9MDwM&tbnid=dJLbrcnNUAc39M:&w=437&h=500&ei=YuJPU5m4BImy0QWDnoCICg&ved=0CAIQxiAwAA&iact=c) |
| 22 | Method of reliability check described and justified? YES - 25% of transcripts were double coded to ensure reliability | [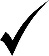](https://www.google.co.uk/imgres?imgurl=http://www.clipartbest.com/cliparts/jRT/Gnk/jRTGnkEiL.png&imgrefurl=http://www.clipartbest.com/check-symbol-in-powerpoint&docid=68WbSLWcx9MDwM&tbnid=dJLbrcnNUAc39M:&w=437&h=500&ei=YuJPU5m4BImy0QWDnoCICg&ved=0CAIQxiAwAA&iact=c) |
| 23 | Did an independent analyst review data and contest themes? - NO | [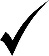](https://www.google.co.uk/imgres?imgurl=http://www.clipartbest.com/cliparts/jRT/Gnk/jRTGnkEiL.png&imgrefurl=http://www.clipartbest.com/check-symbol-in-powerpoint&docid=68WbSLWcx9MDwM&tbnid=dJLbrcnNUAc39M:&w=437&h=500&ei=YuJPU5m4BImy0QWDnoCICg&ved=0CAIQxiAwAA&iact=c) |
| 24 | How were disagreements resolved? Disagreements were resolved through discussion. | [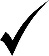](https://www.google.co.uk/imgres?imgurl=http://www.clipartbest.com/cliparts/jRT/Gnk/jRTGnkEiL.png&imgrefurl=http://www.clipartbest.com/check-symbol-in-powerpoint&docid=68WbSLWcx9MDwM&tbnid=dJLbrcnNUAc39M:&w=437&h=500&ei=YuJPU5m4BImy0QWDnoCICg&ved=0CAIQxiAwAA&iact=c) |
|  | Discussion and presentation |  |
| 25 | Findings presented with reference to existing theoretical and empirical literature, and how they contribute - YES | [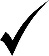](https://www.google.co.uk/imgres?imgurl=http://www.clipartbest.com/cliparts/jRT/Gnk/jRTGnkEiL.png&imgrefurl=http://www.clipartbest.com/check-symbol-in-powerpoint&docid=68WbSLWcx9MDwM&tbnid=dJLbrcnNUAc39M:&w=437&h=500&ei=YuJPU5m4BImy0QWDnoCICg&ved=0CAIQxiAwAA&iact=c) |
| 26 | Strengths and limitations explicitly described and discussed - YES | [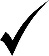](https://www.google.co.uk/imgres?imgurl=http://www.clipartbest.com/cliparts/jRT/Gnk/jRTGnkEiL.png&imgrefurl=http://www.clipartbest.com/check-symbol-in-powerpoint&docid=68WbSLWcx9MDwM&tbnid=dJLbrcnNUAc39M:&w=437&h=500&ei=YuJPU5m4BImy0QWDnoCICg&ved=0CAIQxiAwAA&iact=c) |
| 27 | Evidence of following guidelines (format, word count) – YES - Instructions for authors followed. | [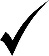](https://www.google.co.uk/imgres?imgurl=http://www.clipartbest.com/cliparts/jRT/Gnk/jRTGnkEiL.png&imgrefurl=http://www.clipartbest.com/check-symbol-in-powerpoint&docid=68WbSLWcx9MDwM&tbnid=dJLbrcnNUAc39M:&w=437&h=500&ei=YuJPU5m4BImy0QWDnoCICg&ved=0CAIQxiAwAA&iact=c) |
| 28 | Detail of methods or additional quotes contained in appendix – NO, but additional quotes are presented in a table (which could be included as an appendix, at the editor’s discretion) | [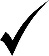](https://www.google.co.uk/imgres?imgurl=http://www.clipartbest.com/cliparts/jRT/Gnk/jRTGnkEiL.png&imgrefurl=http://www.clipartbest.com/check-symbol-in-powerpoint&docid=68WbSLWcx9MDwM&tbnid=dJLbrcnNUAc39M:&w=437&h=500&ei=YuJPU5m4BImy0QWDnoCICg&ved=0CAIQxiAwAA&iact=c) |
| 29 | Written for a health sciences audience - YES | [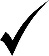](https://www.google.co.uk/imgres?imgurl=http://www.clipartbest.com/cliparts/jRT/Gnk/jRTGnkEiL.png&imgrefurl=http://www.clipartbest.com/check-symbol-in-powerpoint&docid=68WbSLWcx9MDwM&tbnid=dJLbrcnNUAc39M:&w=437&h=500&ei=YuJPU5m4BImy0QWDnoCICg&ved=0CAIQxiAwAA&iact=c) |
| 30 | Are red flags present? – NO. | [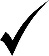](https://www.google.co.uk/imgres?imgurl=http://www.clipartbest.com/cliparts/jRT/Gnk/jRTGnkEiL.png&imgrefurl=http://www.clipartbest.com/check-symbol-in-powerpoint&docid=68WbSLWcx9MDwM&tbnid=dJLbrcnNUAc39M:&w=437&h=500&ei=YuJPU5m4BImy0QWDnoCICg&ved=0CAIQxiAwAA&iact=c) |
