# Supplementary material for: Conceptualising patient empowerment: a mixed methods study
Source: BMC Health Serv Res. 2015 Jul 1;15:252. doi: 10.1186/s12913-015-0907-z (PMC4488113; doi:10.1186/s12913-015-0907-z)
Supplement: Additional file 2: — Details of included articles. [file 12913_2015_907_MOESM2_ESM.docx]

**Supplementary Information: Details of included articles**

| **AUTHOR/YEAR** | **COUNTRY** | **PURPOSE OF STUDY** | **METHODOLOGICAL APPROACH**  **1 = quantitative, 2 = qualitative, 3 = mixed methods, 4= Other** |
| --- | --- | --- | --- |
| Alegria et al 2008(1) | US | This study developed and evaluated a patient self-reported activation and empowerment strategy in mental health care | 2 |
| Alpay 2010(2) | The Netherlands | To propose directions for best practices, with the aim of developing e-health tools that further patient empowerment | 4 Literature Review |
| Ammenwerth 2011(3) | N/A | To review the impact of electronic patient portals on patient empowerment | 4 Literature Review |
| Anderson 2010(4) | N/A | To clarify the concept of empowerment and to correct common misconceptions about its use in diabetes care and education | 4 Essay |
| Asimakopoulou 2012(5) | N/A | N/A | 4 Commentary |
| Asimakopoupou 2012(6) | UK | This exploratory study examines what Health Care Professionals working with diabetes patients, understand by the term ‘empowerment’, their attitudes towards it and whether they believe they practise in ways consistent with empowerment principles | 2 |
| Aujoulat 2008(7) | Belgium and Italy | To understand the process of empowerment as it may occur in patients whose experience of illness has at some point induced a feeling of powerlessness | 2 |
| Ayme 2008(8) | N/A | N/A | 4 Essay |
| Baars 2009(9) | The Netherlands | To assess inflammatory bowel disease patients’ perceptions of therapy adherence and disease related functional status in members of the Dutch patients’ association of Crohn’s disease and ulcerative colitis | 1 |
| Baars 2010(10) | The Netherlands | To assess inflammatory bowel disease patients’ preferences about being involved in such decisions | 1 |
| Bann 2010(11) | US | To develop and psychometrically evaluate scales to measure patients’ perceptions of provider support, patient-centred care, and empowerment as predictors of health outcomes. | 3 |
| Barrie 2011(12) | N/A | N/A | 4 Essay |
| Bartlett 2011(13) | Not specified – online participants | To investigate which empowerment processes and outcomes were present and if and/or how they affected the doctor/patient relationship | 1 |
| Batifoulier 2011(14) | France | To explore the way economic policy has sought to encourage active, well-informed patients by giving them market power | 4 Essay |
| Begum 2010(15) | UK | To critically analyse the impact of National Service Framework policy and its effects on health care | 4 Essay |
| Bos 2008(16) | N/A | To show the implication of interactive ICT on patient empowerment, through an overview of some of the key aspects - EHR, telecare and patient networks - all this within the context of recent Health 2.0 developments. | 4 Essay |
| Boudioni 2012(17) | Greece and UK | To explore and compare the general patient empowerment settings in the two countries | 3 |
| Brown 2012(18) | N/A | N/A | 4 Essay |
| Brugge 2009(19) | US | To elicit discussion about patient-provider communication, particularly with respect to the concerns of the health literacy framework | 2 |
| Bruggers 2012(20) | N/A | N/A | 4 Essay |
| Buccoliero 2010(21) | Italy | To provide an analysis of the key factors of an efficient web strategy for healthcare organisations with regards to the issue of patient empowerment | 1 |
| Buchner 2009(22) | N/A | N/A | 4 Editorial |
| Callan 2011(23) | N/A | N/A | 4 Editorial |
| Camerini 2012(24) | Switzerland | To present an empirical, a priori, cross-sectional evaluation of the differential effects of health knowledge and empowerment on patients’ self-management and health outcomes. | 1 |
| Chang 2012(25) | Korea | To compare the effect of a nurse-led empowerment-based intervention to that of standard care on metabolic syndrome risk factors, self-management behaviours, and walking activity in Korean hypertensive patients. | 1 |
| Chung 2012(26) | N/A | N/A | 4 Essay |
| Falcao-Reis 2010(27) | Portugal | To present a patient's privacy generic control mechanisms scenarios based on the Extended OpenID | 4 Essay |
| Funnel 2008(28) | N/A | To outline four fundamental lessons that need to be addressed as part of patient education and provides a simple approach called the LIFE plan to help patients truly take charge of their diabetes. | 4 Essay |
| Groene 2010(29) | Europe/ Multi-countries | To study the relationships among organisational quality improvement systems, patient empowerment, organisational culture, professionals’ involvement with the quality of hospital care, including clinical effectiveness, patient safety and patient involvement | 3 |
| Hagiwara 2011(30) | N/A | To analyse Japanese and foreign papers related to the empowerment of cancer patients, and to identify trends and issues related to studies on cancer patient empowerment | 4 Literature Review |
| Hiley 2008(31) | UK | To evaluate the introduction of a new type of syringe for patients on a self-injection of methotrexate (MTX) programme and explore patients’ sense of empowerment relating to the self-injection of MTX. | 3 |
| Holmström 2010(32) | N/A | To explore and compare the concepts of patient-centeredness and patient empowerment, and clarify a possible relationship between the two from the perspective of the encounter between patients and their healthcare providers. | 4 Literature Review |
| LaVista 2009(33) | US | To develop and evaluate an educational video to empower patients and their families to initiate a conversation with their physician on the benefits and risks of hydroxyurea encouraging a shared decision making process | 1 |
| Lejbokowicz 2012(34) | N/A | N/A | 4 Essay |
| Lo 2012(35) | N/A | N/A | 4 Editorial |
| Louie 2012(36) | China | To compare the effects of a patient and carer empowerment programme and conventional hip fracture protocol | 1 |
| McAllister 2008(37) | UK | To develop a theoretical framework describing the patient benefits from using clinical genetics services. | 2 |
| McAllister 2011(38) | UK | To (1) explore the validity, relevance and importance of the Empowerment construct and refine the construct as necessary and (2) inform the decision whether or not to develop a clinical genetics-specific PROM of Empowerment. | 2 |
| McAllister 2012(39) | UK | To argue for consideration of patient empowerment itself as a directly measurable patient reported outcome for chronic conditions, highlight some issues in adopting this approach, and outline a research agenda to enable healthcare evaluation on the basis of patient empowerment. | 4 Essay |
| McCarley 2009(40) | N/A | N/A | 4 Essay |
| McGuckin 2011(41) | N/A | To present the evidence from the current literature supporting the use of programs that aim to encourage patients to take a more active role in their care, especially with regard to hand hygiene promotion. | 4 Literature Review |
| Meyer 2008(42) | Germany | To investigate the impact of clinical research for the empowerment of our patients | 2 |
| Moattari 2012(43) | Iran | To determine the impact of an empowerment program on self-efficacy, quality of life, clinical indicators of blood pressure and interdialytic weight gain, and laboratory results in these patients. | 1 |
| Mola 2008(44) | N/A | To show that many characteristics of general practice are already oriented towards patient empowerment. | 4 Literature Review |
| Musacchio 2011(45) | Italy | To document the impact of the first 2 years of SINERGIA on clinical outcomes of patients and on routine practice of the clinic | 1 |
| Nygardh 2011(46) | Sweden | To explore empowerment in outpatient care as experienced by these family members | 2 |
| Nygardh 2012(47) | Sweden | To explore empowerment within the patient–staff encounter as experienced by out-patients with chronic kidney disease. | 2 |
| Oh 2012(48) | Korea | To test a mechanism through which Korean diabetes patients’ exchange of computer-mediated social support (CMSS) in diabetes online communities influences their sense of empowerment and intention to actively communicate with the doctor. | 1 |
| Pagliarello 2010(49) | Italy | To develop a tool for measuring levels of empowerment in psoriatic patients, and to assess its validity and reliability. | 1 |
| Pagliarello 2011(50) | Italy | To evaluate an educational programme for increasing empowerment, in a spa setting | 1 |
| The Lancet 2012(51) | N/A | N/A | 4 Editorial |
| Petersen 2008(52) | Denmark | To describe the methods employed in the Hillerød Programme | 4 Essay |
| Piper 2010(53) | UK | To describe the meaning of the theme of empowerment from research on health promotion in nursing from the perspective of nurses participating in the study | 2 |
| Quantin 2011(54) | N/A | To point out the unavoidable empowerment of patients with regard to their personal health record and propose the mixed management of patients' medical records. | 4 Essay |
| Samoocha 2010(55) | N/A | To evaluate whether Web-based interventions are effective in increasing patient empowerment compared with usual care or face-to-face interventions. | 4 Literature Review |
| Schulz 2011(56) | N/A | N/A | 4 Essay |
| Schulz 2013(57) | N/A | To argue that health literacy and patient empowerment are distinct concepts but closely interwoven and must be considered in conjunction to understand individual health behavior and the impact on it of communications | 4 Literature Review |
| Suter 2011(58) | N/A | To describe how remote monitoring, in combination with the application of salient adult learning and cognitive behavioral theories and applied to telehealth care delivery and practice, can promote improved patient self-efficacy with disease management | 4 Essay |
| Tol 2012(59) | Iran | To assess the empowerment score among diabetic patients | 1 |
| Topac 2011(60) | Romania | To describe how telecare system can become more friendly | 4 Essay |
| van den Berg 2010(61) | UK | To investigate the depiction of the patient–pharmacist power relationship within MUR patient information leaflets. | 2 |
| van Uden-Kraan 2008(62) | The Netherlands | To explore if lurkers in online patient support groups profit to the same extent as posters do | 1 |
| van Uden-Kraan 2009(63) | The Netherlands | To explore, by means of a questionnaire, to what extent patients feel empowered by their participation in online support groups and with which frequency empowering processes occur | 1 |
| Wahlin 2009(64) | Sweden | To compare intensive care units patient experiences of empowerment with next of kin and staff beliefs | 2 |
| Wan 2012(65) | US | To explore how patients in an urban, public-sector clinic perceive patient empowerment as it applies to their treatment, interactions with clinicians, and self-care behaviors. | 2 |
| Wentzer 2010(66) | The Netherlands | To discuss whether online support groups in patient treatment are to be understood in the light of patient empowerment or within the tradition of compliance | 3 |
| Yin 2012(67) | US | To describe a prototype of a serious game designed to improve hospital patients’ confidence in managing their hospital stay. | 3 |

1. Alegria M, Polo A, Gao S, Santana L, Rothstein D, Jimenez A, et al. Evaluation of a patient activation and empowerment intervention in mental health care. Medical Care. 2008;46(3):247-56.

2. Alpay LL, Henkemans OB, Otten W, Rovekamp TA, Dumay AC. E-health applications and services for patient empowerment: Directions for best practices in the Netherlands. Telemedicine and e-Health. 2010;16(7):787-91.

3. Ammenwerth E, Schnell-Inderst P, Hoerbst A. Patient empowerment by electronic health records: first results of a systematic review on the benefit of patient portals. Studies in Health Technology & Informatics. 2011;165:63-7.

4. Anderson RM, Funnell MM. Patient empowerment: myths and misconceptions. Patient Education & Counseling. 2010;79(3):277-82.

5. Asimakopoulou K, Gilbert D, Newton P, Scambler S. Back to basics: Re-examining the role of patient empowerment in diabetes. Patient Education & Counseling. 2012;86(3):281-3.

6. Asimakopoulou K, Newton P, Sinclair AJ, Scambler S. Health care professionals' understanding and day-to-day practice of patient empowerment in diabetes; time to pause for thought? Diabetes Research & Clinical Practice. 2012;95(2):224-9.

7. Aujoulat I, Marcolongo R, Bonadiman L, Deccache A. Reconsidering patient empowerment in chronic illness: A critique of models of self-efficacy and bodily control. Social Science & Medicine. 2008;66(5):1228-39.

8. Aymé S, Kole A, Groft S. Empowerment of patients: lessons from the rare diseases community. The Lancet. 2008;371(9629):2048-51.

9. Baars JE, Zelinkova Z, Mensink PBF, Markus T, Looman CWN, Kuipers EJ, et al. High therapy adherence but substantial limitations to daily activities amongst members of the Dutch inflammatory bowel disease patients' organization: A patient empowerment study. Alimentary Pharmacology and Therapeutics. 2009;30(8):864-72.

10. Baars JE, Markus T, Kuipers EJ, Van Der Woude CJ. Patients' preferences regarding shared decision-making in the treatment of inflammatory bowel disease: Results from a patient-empowerment study. Digestion. 2010;81(2):113-9.

11. Bann CM, Sirois FM, Walsh EG. Provider support in complementary and alternative medicine: exploring the role of patient empowerment. Journal of Alternative & Complementary Medicine. 2010;16(7):745-52.

12. Barrie J. Patient empowerment and choice in chronic pain management. Nursing Standard. 2011;25(31):38-41.

13. Bartlett YK, Coulson NS. An investigation into the empowerment effects of using online support groups and how this affects health professional/patient communication. Patient Education and Counseling. 2011;83(1):113-9.

14. Batifoulier P, Domin JP, Gadreau M. Market empowerment of the patient: The French experience. Review of Social Economy. 2011;69(2):143-62.

15. Begum S, Por J. The impact of the NSF for Diabetes on patient empowerment. British Journal of Nursing. 2010;19(14):887-90.

16. Bos L, Marsh A, Carroll D, Gupta S, Rees M, editors. Patient 2.0 empowerment2008.

17. Boudioni M, McLaren SM, Lister G. Cross-national diagnostic analysis of patient empowerment in England and Greece. International Journal of Caring Sciences. 2012;5(3):246-63.

18. Brown C, Bornstein E, Wilcox C. Partnership and empowerment program: A model for patient-centered, comprehensive, and cost-effective care. Clinical Journal of Oncology Nursing. 2012;16(1):15-7.

19. Brugge D, Edgar T, George K, Heung J, Laws MB. Beyond literacy and numeracy in patient provider communication: Focus groups suggest roles for empowerment, provider attitude and language. BMC Public Health. 2009;9.

20. Bruggers CS, Altizer RA, Kessler RR, Caldwell CB, Coppersmith K, Warner L, et al. Patient-empowerment interactive technologies. Science Translational Medicine. 2012;4(152).

21. Buccoliero L, Bellio E, Prenestini A. Patient Web Empowerment Index (PWEI): an index for assessment of healthcare providers' Web strategies. Case study: PPEI application in Italy... MEDINFO 2010: Proceedings of the 13th World Congress on Medical Informatics, Part 1. Studies in Health Technology & Informatics. 2010;160:38-42.

22. Buchner B. Editorial: The pharmaceutical package of the European commission: Empowerment for patients? European Journal of Health Law. 2009;16(3):201-6.

23. Callan CM. Patient empowerment and multimodal hand hygiene promotion: a win-win strategy. American Journal of Medical Quality. 2011;26(1):6-7.

24. Camerini L, Schulz PJ, Nakamoto K. Differential effects of health knowledge and health empowerment over patients' self-management and health outcomes: A cross-sectional evaluation. Patient Education and Counseling. 2012;89(2):337-44.

25. Chang AK, Fritschi C, Kim MJ. Nurse-led empowerment strategies for hypertensive patients with metabolic syndrome. Contemporary Nurse. 2012;42(1):118-28.

26. Chung R. Telehealth physician oversight over direct to consumer testing: Doctors working with patients towards patient empowerment. Telemedicine and e-Health. 2012;18(5):354-9.

27. Falcao-Reis F, Correia ME. Patient empowerment by the means of citizen-managed Electronic Health Records: web 2.0 health digital identity scenarios. Studies in Health Technology & Informatics. 2010;156:214-28.

28. Funnell MM, Weiss MA. Patient empowerment: the LIFE approach. European Diabetes Nursing. 2008;5(2):75-8.

29. Groene O, Klazinga N, Wagner C, Arah OA, Thompson A, Bruneau C, et al. Investigating organizational quality improvement systems, patient empowerment, organizational culture, professional involvement and the quality of care in European hospitals: the 'Deepening our Understanding of Quality Improvement in Europe (DUQuE)' project. BMC Health Services Research. 2010;10:281-.

30. Hagiwara E, Futawatari T. Trends and issues related to studies on cancer patient empowerment. Kitakanto Medical Journal. 2011;61(3):367-75.

31. Hiley J, Homer D, Clifford C. Patient self-injection of methotrexate for inflammatory arthritis: A study evaluating the introduction of a new type of syringe and exploring patients' sense of empowerment. Musculoskeletal Care. 2008;6(1):15-30.

32. Holmström I, Röing M. The relation between patient-centeredness and patient empowerment: a discussion on concepts. Patient Education & Counseling. 2010;79(2):167-72.

33. La Vista JM, Treise DM, Dunbar LN, Ritho J, Hartzema AG, Lottenberg R. Development and evaluation of a patient empowerment video to promote hydroxyurea adoption in sickle cell disease. Journal of the National Medical Association. 2009;101(3):251-7.

34. Lejbkowicz I, Caspi O, Miller A. Participatory medicine and patient empowerment towards personalized healthcare in multiple sclerosis. Expert Review of Neurotherapeutics. 2012;12(3):343-52.

35. Lo YYC. Patient empowerment. Hong Kong Practitioner. 2012;34(3):89-91.

36. Louie SWS, Poon MY, Yu SY, Chan WL, Au KM, Wong KM. Effectiveness of a patient/carer empowerment programme for people with hip fractures. International Journal of Therapy and Rehabilitation. 2012;19(12):673-81.

37. McAllister M, Payne K, MacLeod R, Nicholls S, Donnai D, Davies L. Patient empowerment in clinical genetics services. Journal of Health Psychology. 2008;13(7):895-905.

38. McAllister M, Dunn G, Todd C. Empowerment: Qualitative underpinning of a new clinical genetics-specific patient-reported outcome. European Journal of Human Genetics. 2011;19(2):125-30.

39. McAllister M, Dunn G, Payne K, Davies L, Todd C. Patient empowerment: the need to consider it as a measurable patient-reported outcome for chronic conditions. BMC Health Services Research. 2012;12:157.

40. McCarley P. Patient empowerment and motivational interviewing: engaging patients to self-manage their own care. Nephrology Nursing Journal. 2009;36(4):409-13.

41. McGuckin M, Storr J, Longtin Y, Allegranzi B, Pittet D. Patient empowerment and multimodal hand hygiene promotion: a win-win strategy. American Journal of Medical Quality. 2011;26(1):10-7.

42. Meyer C, Muhlfeld A, Drexhage C, Floege J, Goepel E, Schauerte P, et al. Clinical research for patient empowerment--a qualitative approach on the improvement of heart health promotion in chronic illness. Medical Science Monitor. 2008;14(7):CR358-65.

43. Moattari M, Ebrahimi M, Sharifi N, Rouzbeh J. The effect of empowerment on the self-efficacy, quality of life and clinical and laboratory indicators of patients treated with hemodialysis: A randomized controlled trial. Health and Quality of Life Outcomes. 2012;10.

44. Mola E, De Bonis JA, Giancane R. Integrating patient empowerment as an essential characteristic of the discipline of general practice/family medicine. European Journal of General Practice. 2008;14(2):89-94.

45. Musacchio N, Lovagnini Scher A, Giancaterini A, Pessina L, Salis G, Schivalocchi F, et al. Impact of a chronic care model based on patient empowerment on the management of Type2 diabetes: Effects of the SINERGIA programme. Diabetic Medicine. 2011;28(6):724-30.

46. Nygårdh A, Wikby K, Malm D, Ahlstrom G. Empowerment in outpatient care for patients with chronic kidney disease - from the family member's perspective. BMC Nursing. 2011;10.

47. Nygårdh A, Malm D, Wikby K, Ahlström G. The experience of empowerment in the patient-staff encounter: The patient's perspective. Journal of Clinical Nursing. 2012;21(5-6):897-904.

48. Oh HJ, Lee B. The effect of computer-mediated social support in online communities on patient empowerment and doctor-patient communication. Health Communication. 2012;27(1):30-41.

49. Pagliarello C, Di Pietro C, Paradisi A, Abeni D, Tabolli S. Measuring empowerment in patients with psoriasis: The psoriasis empowerment enquiry in the routine practice (PEER) questionnaire. European Journal of Dermatology. 2010;20(2):200-4.

50. Pagliarello C, Calza A, Armani E, di Pietro C, Tabolli S. Effectiveness of an empowerment-based intervention for psoriasis among patients attending a medical spa. European Journal of Dermatology. 2011;21(1):62-6.

51. Patient empowerment--who empowers whom? Lancet. 2012;379(9827):1677-.

52. Petersen SK, Tribler J, Mølsted S. Empowerment-inspired patient education in practice and theory. European Diabetes Nursing. 2008;5(3):99-103.

53. Piper S. Patient empowerment: emancipatory or technological practice? Patient Education & Counseling. 2010;79(2):173-7.

54. Quantin C, Benzenine E, Auverlot B, Jaquet-Chiffelle DO, Coatrieux G, Allaert FA, editors. Empowerment of patients over their personal health record implies sharing responsibility with the physician2011.

55. Samoocha D, Bruinvels DJ, Elbers NA, Anema JR, Aj. Effectiveness of Web-based interventions on patient empowerment: a systematic review and meta-analysis. Journal of Medical Internet Research. 2010;12(2):e23-e.

56. Schulz PJ, Nakamoto K, editors. "Bad" literacy, the internet, and the limits of patient empowerment2011.

57. Schulz PJ, Nakamoto K. Health literacy and patient empowerment in health communication: The importance of separating conjoined twins. Patient Education and Counseling. 2013;90(1):4-11.

58. Suter P, Suter WN, Johnston D. Theory-Based Telehealth and Patient Empowerment. Population Health Management. 2011;14(2):87-92.

59. Tol A, Sharifirad G, Shojaeezadeh D, Alhani F, Tehrani MM. Determination of empowerment score in type 2 diabetes patients and its related factors. Journal of the Pakistan Medical Association. 2012;62(1).

60. Topac V, Stoicu-Tivadar V. Patient empowerment by increasing information accessibility in a telecare system. Studies in Health Technology & Informatics. 2011;169:681-5.

61. van den Berg M, Donyai P. How was patient empowerment portrayed in information leaflets describing the community pharmacy Medicines Use Review service in the UK? Patient Education and Counseling. 2010;80(2):274-6.

62. van Uden-Kraan CF, Drossaert CH, Taal E, Seydel ER, van de Laar MA. Self-reported differences in empowerment between lurkers and posters in online patient support groups. Journal of Medical Internet Research. 2008;10(2).

63. van Uden-Kraan CF, Drossaert CHC, Taal E, Seydel ER, Maf. Participation in online patient support groups endorses patients' empowerment. Patient Education & Counseling. 2009;74(1):61-9.

64. Wåhlin I, Ek AC, Idvall E. Empowerment in intensive care: Patient experiences compared to next of kin and staff beliefs. Intensive and Critical Care Nursing. 2009;25(6):332-40.

65. Wan CR, Vo L, Barnes CS. Conceptualizations of patient empowerment among individuals seeking treatment for diabetes mellitus in an urban, public-sector clinic. Patient Education and Counseling. 2012;87(3):402-4.

66. Wentzer H, Bygholm A. Compliance or patient empowerment in online communities: reformation of health care services? Studies in Health Technology & Informatics. 2010;157:141-7.

67. Yin L, Ring L, Bickmore T, editors. Using an interactive visual novel to promote patient empowerment through engagement2012.
